# CEUS LI-RADS: a pictorial review

**DOI:** 10.1186/s13244-019-0819-2

**Published:** 2020-02-04

**Authors:** Tommaso Vincenzo Bartolotta, Maria Chiara Terranova, Cesare Gagliardo, Adele Taibbi

**Affiliations:** 1 BiND Department: Biomedicine, Neuroscience and Advanced Diagnostic, University of Palermo, Via Del Vespro, 129 90127 Palermo, Italy; 2 Department of Radiology, Fondazione Istituto Giuseppe Giglio Ct.da Pietrapollastra, Via Pisciotto, 90015 Cefalù (Palermo), Italy

**Keywords:** Contrast-enhanced ultrasonography, Hepatocellular carcinoma, Cholangiocarcinoma, Liver tumor characterization, Cirrhosis

## Abstract

Contrast-enhanced ultrasound (CEUS) greatly improved the diagnostic accuracy of US in the detection and characterization of focal liver lesions (FLLs), and it is suggested and often included in many international guidelines as an important diagnostic tool in the imaging work-up of cirrhotic patients at risk for developing hepatocellular carcinoma (HCC). In particular, CEUS Liver Imaging Reporting and Data System (LI-RADS) provides standardized terminology, interpretation, and reporting for the diagnosis of HCC. The aim of this pictorial essay is to illustrate CEUS features of nodules discovered at US in cirrhotic liver according to LI-RADS categorization.

## Key points

- CEUS is a safe, robust, and cost-effective imaging modality
- CEUS allows in real time a confident characterization of hepatocellular carcinoma (HCC)
- CEUS LI-RADS provides standardized terminology, interpretation, and reporting for the diagnosis of HCC

## Introduction

Worldwide HCC is reported to be the sixth most common tumor and the fourth cause of death related to cancer [1].

Cirrhotic patients are particularly considered a high-risk group for the onset of HCC, prompting several international scientific societies to publish guidelines recommending surveillance of adults with cirrhosis on the evidence of improved overall survival [2–4]. The recommended imaging surveillance tool for early detection of HCC is ultrasound (US), usually performed every 6 months [2, 3]. Once a new nodule suspect for HCC is discovered in the liver of a cirrhotic patient, further imaging work-up with either computed tomography (CT) or magnetic resonance imaging (MRI) is usually recommended [2, 3]. Both techniques require the intravenous administration of contrast media and difficulties may arise in patients with severe renal failure or allergies [5, 6].

Contrast-enhanced US (CEUS) allows to assess non-invasively the contrast enhancement patterns of HCC, without the use of ionizing radiation and with a much higher temporal resolution than CT and MRI [7–10]. This unique feature of CEUS virtually eliminates the possibility of image acquisition mistiming, especially in the arterial phase [11]. Several studies have reported the improvement in diagnostic accuracy of CEUS in the detection and characterization of FLLs, including HCC [8–11]. CEUS has been also proved useful in the guidance and response assessment of therapeutic procedures [12–18].

CEUS examination is performed by injecting intravenously microbubble-based contrast agents (USCAs) consisting of flexible shells (e.g., phospholipids, liposomes) presenting a radius ranging from 1 to 10 μm, containing low solubility gases (e.g., perfluoropropane, perfluorocarbon, or sulphur hexafluoride) [19, 20]. USCAs microbubbles pass through the lung capillary bed and they remain confined within the intravascular space. Although some USCAs may present a post-vascular phase in the liver and spleen, this phase is currently not taken in account for the characterization of HCC [21]. Approximately 20 min after the injection, the USCAs are completely eliminated: the gas diffuses into the blood and then exhaled via the pulmonary route, while the shell components are metabolized by the liver or filtered by the kidney [22].

USCAs are generally safe and well tolerated with a safety profile better than or similar to CT and MRI contrast media [23]. They are not nephrotoxic and may be used even in patients with severe renal failure, renal obstruction, or chronic obstructive pulmonary disease. Hence, there is no need of laboratory tests for assessing renal function before administering USCAs.

Currently, in many clinical settings, CEUS is recommended as pivotal imaging tool in the diagnostic work-up of liver FLLs, including HCC, also considering its favorable cost-benefit ratio when compared with cross-sectional imaging techniques [24–27]. In a recent meta-analysis, CEUS showed excellent diagnostic accuracy in differentiating malignant from benign FLLs with pooled sensitivity of 0.92, pooled specificity of 0.87, and diagnostic odds ratio of 104.20 respectively [21].

The CEUS cases presented in this paper were acquired by means of various ultrasound equipments: RS80A and RS85, (Samsung Medison, Co. Ltd.), iU22 (Philips Ultrasound, Bothell, Wash, USA), and MyLab Twice (Esaote, Genova, Italy). All of these units were provided with multifrequency convex array probes and contrast-specific imaging software.

### CEUS LI-RADS

#### CEUS LI-RADS system

Firstly released in 2016 by the ACR and then revised in 2017, contrast enhanced ultrasound (CEUS) Liver Imaging Reporting and Data System (LI-RADS) is a standardized system for technique, interpretation, reporting, and data collection for CEUS exams in patients at risk for developing HCC [28].

CEUS LI-RADS lexicon integrates with the previously released CT/MRI LI-RADS lexicons, and it is intended to allow the radiologists to (1) use consistent terminology, (2) reduce variability and mistakes in imaging interpretation, (3) promote communication with referring clinicians, and (4) facilitate research and quality assurance [29].

Noteworthy, although FLL characterization features using CEUS are similar to those of multiphasic CT and/or MRI, there are still important differences between these techniques, regarding both features and characterization algorithm [30]. CEUS LI-RADS is intended for the use with purely intravascular microbubble contrast agents—such as Lumason® (in USA)/SonoVue® (outside USA) and Definity® (in USA, Canada)/Luminity® (outside USA, Canada)—which affects washout and “capsule” characterization [11, 31, 32]. Actually, CEUS washout is true washout. On the other hands, CEUS does not depict “capsule”; hence, “capsule” is not a CEUS major feature. CEUS usually does not depict vascular pseudo-lesions such as arterioportal shunts, a frequent cause of diagnostic confusion on CT and MRI: as consequence, any CEUS enhancing observation is a true lesion [33].

Of note, the use of a combined blood pool and Kupffer cell agent (Sonazoid®) is not contemplated in FLL characterization using CEUS LI-RADS [28].

#### When is CEUS LI-RADS categorization system indicated?

CEUS LI-RADS must be applied only in patients at high risk for developing HCC (cirrhosis, chronic hepatitis B, current or prior HCC, adult liver transplant candidates, and recipients after transplant). CEUS LI-RADS must not be applied to patients without the abovementioned risk factors or < 18 years old. Table 1 lists the man indications of CEUS LI-RADS in patients at high risk for HCC [28].

**Table 1:** Main indications for CEUS LI-RADS in patients at high risk for HCC

Assess nodules ≥ 10 mm detected on surveillance US.	
Assess LR-3, LR-4, and LR-M observations detected on prior CT or MRI.	
Detect APHE when mistiming is suspected as the reason for its absence on prior CT or MRI.	
Assess biopsied observations with inconclusive histology.	
Guide biopsy or treatment of observations difficult to visualize with precontrast US.	
Help select appropriate observation(s) or observation component(s) for biopsy.	
Monitor changes in enhancement pattern over time for selected CEUS LR-3 or CEUS LR-4 observations.	
Differentiate tumor in vein (“tumor thrombus”) from bland thrombus.	

### CEUS LI-RADS reporting and categories

According to CEUS LI-RADS criteria, the two major features of HCC are (1) arterial phase enhancement (not rim or globular peripheral) and (2) washout. Not surprisingly, CEUS sensitivity in the observation of arterial hypervascularity from nodules in liver cirrhosis has been showed to be significantly higher than that of CT/MRI [34–36].

#### Washout

Washout is defined as a reduction in enhancement in whole or in part in comparison with the liver resulting in hypoenhancement. This latter may begin during or after arterial phase. Furthermore, CEUS characterization of washout requires assessment of its onset (late vs. early) and degree (mild vs. marked), not just its presence. Actually, early (< 60 s) and/or marked washout is a major feature for LR-M [37]. On the other hand, late (≥ 60 s) and mild washout is a major feature for HCC [38].

The degree of washout is defined “mild” when the nodule enhances less than liver, but not some enhancement persists. If this persistent enhancement disappears after 2 min, the degree of washout is still considered mild, even if the nodule eventually becomes “punched-out.” On the other hand, the degree of washout is defined “marked” when the nodule lacks of any contrast enhancement within 2 min after contrast injection: the observation appears black or “punched out.”

The ancillary imaging features can be taken into account for category adjustment when category classification is not definite and, as stated by ACR, they can be used to upgrade or downgrade unclear FLLs categories. Presence of ancillary features favoring malignancy (size growth, mosaic, and nodule in nodule architecture) can only upgrade by one category (except LR-4 to LR-5) unclear lesions. On the other hand, presence of ancillary features favoring benignity (size stability or size reduction) can only downgrade by one category unclear lesions. When conflicting, it is recommended not to use them to adjust category.

**Fig. 1:**
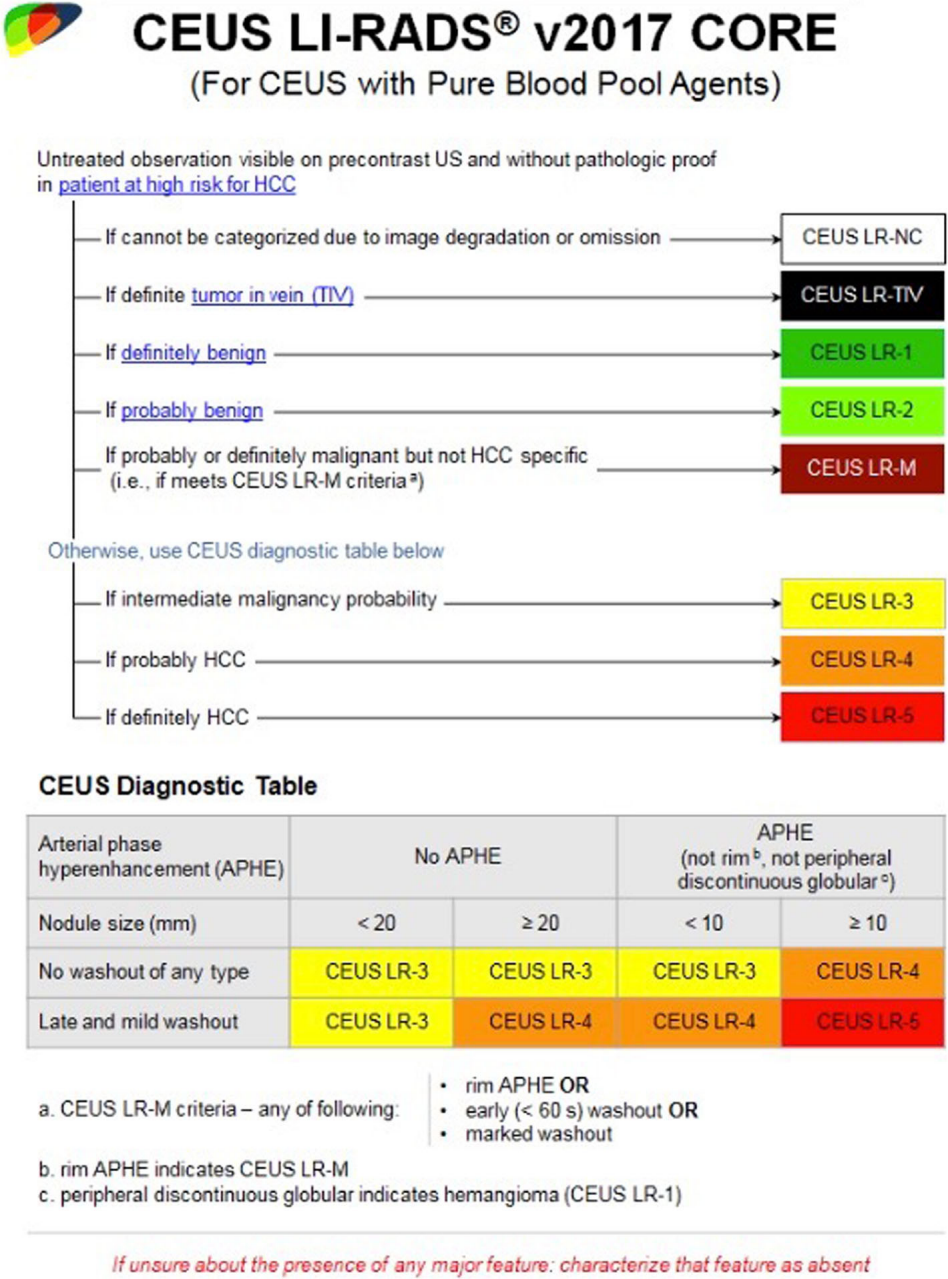
ACR CEUS LI-RADS categorization and diagnostic algorithm

The presence of patent veins or the presence of thrombosis must be assessed as well [28]. Currently, there are eight CEUS LI-RADS diagnostic categories with related imaging work-up suggestions (Fig. 1). In particular, categories from 1 to 5 include nodules with increasing probability of malignancy. Of note, a one-to-one correspondence between such categories and histologic progression or grade of cirrhosis-associated nodules does not exist [39]. As consequence, no cirrhotic nodule is included in LR-1 and many HCCs might be categorized LR-4 or lower. The LR categorization impact on the imaging workup options as well, as described in Table 2.

**Table 2:** CEUS LI-RADS categories

Diagnostic categories	Clinical significance	Imaging workup options		
		Return to routine surveillance	Alternative diagnostic imaging (i.e., CT or MRI)	Repeat CEUS
CEUS LR-NC	Not categorizable (due to image degradation or omission)	–	≤ 3 months*	≤ 3 months**
CEUS LR-1	Definitely benign	6 months**	–	–
CEUS LR-2	Probably benign	6 months**	–	≤ 6 months*
CEUS LR-3	Intermediate probability of malignancy	–	≤ 6 months**	≤ 6 months*
CEUS LR-4	Probably HCC	Multidisciplinary discussion may be needed for consensus management. If neither biopsy nor treatment is planned: repeat or alternative diagnostic imaging in ≤ 3 months		
CEUS LR-5	Definitely HCC	Diagnosis of HCC. Multidisciplinary discussion for consensus management.		
CEUS LR-M	Probably or definitely malignant, not necessarily HCC	Multidisciplinary discussion for consensus management. May include alternative or repeat imaging, biopsy, or treatment		
CEUS LR-TIV	Tumor in vein	Multidisciplinary discussion for consensus management. May include biopsy or biomarker correlation to determine etiology of TIV: HCC, ICC, other.		

**Preferred option in most cases

*Reasonable alternative option. Not recommended

### How to apply CEUS LI-R-RADS system?

If after the injection of microbubble contrast agent, any observation results not assessable due to image degradation or omission, CEUS LR-NC (not categorizable) must be used. In this case, information about the cause technical limitations or artifacts should be reported and further work-up advice should be also provided, such as repeat CEUS or perform alternative imaging modality (i.e., CT and/or MRI) within 3 months (Table 2).

**Fig. 2:**
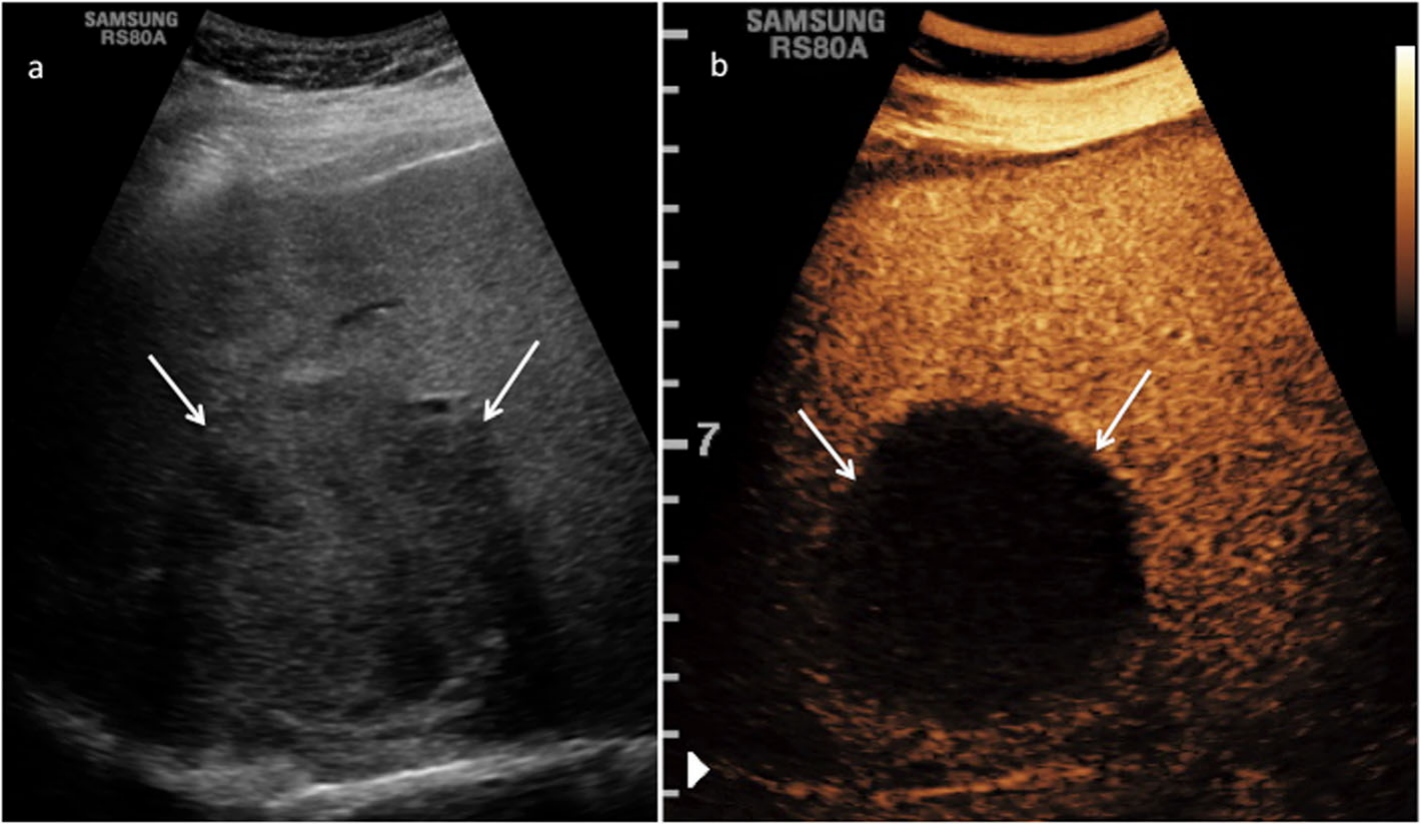
CEUS LI-RADS 1 (definitely benign). Complex cyst in a 60-year-old woman with chronic hepatitis B viral infection. Baseline US image (**a**) shows a heterogeneous and moderately hypoechoic lesion sized 6.2 cm in the segment VI–VII (arrows). At CEUS (**b**), the lesion shows a complete lack of enhancement throughout the vascular phases (arrows)

**Fig. 3:**
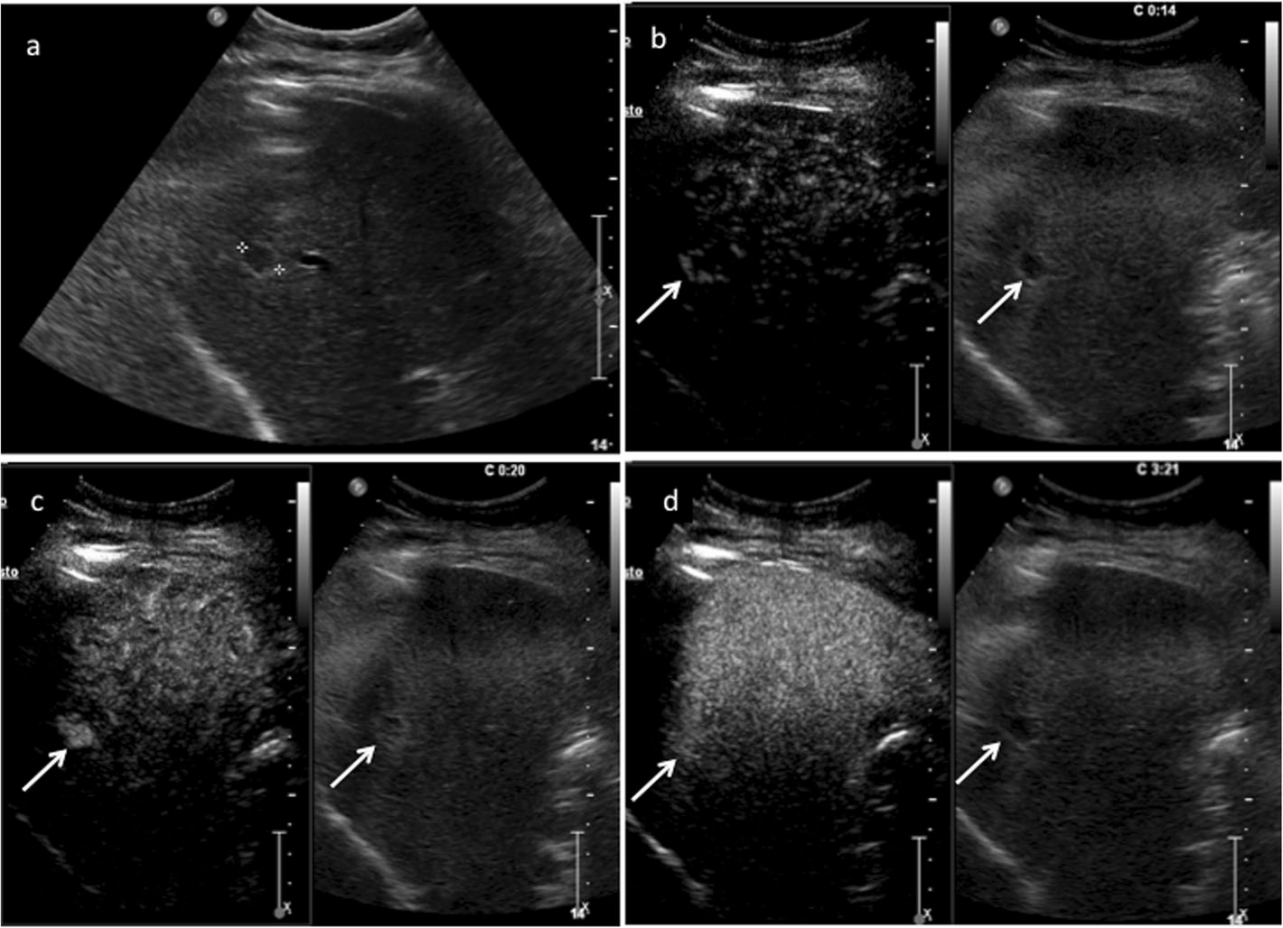
CEUS LI-RADS 1 (definitely benign). Hemangioma in a 49-year-old woman with chronic hepatitis B viral infection. Baseline US image (**a**) shows a hypoechoic lesion surrounded by a tiny peripheral hyperechoic rim sized 1.3 cm in the segment VII (calipers). CEUS in the early arterial phase (**b**) depicts peripheral globular enhancement (arrows) followed by a complete centripetal fill-in in the late arterial phase (**c**) (arrows). The lesion shows sustained contrast-enhancement in the extended portal-venous phase (**d**) (arrows)

**Fig. 4:**
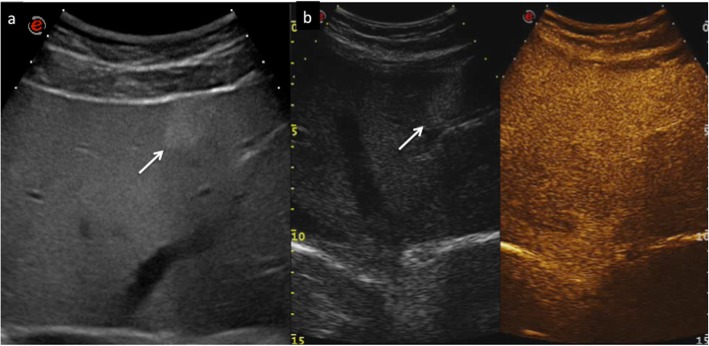
CEUS LI-RADS 2 (probably benign). Atypically located area of hypersteatosis in a 47-year-old woman with chronic hepatitis B viral infection. Baseline US image (**a**) shows a hyperechoic oval-shaped area sized 2.8 cm in the segment IV in a fatty liver (arrow). At CEUS in the extended portal venous phase (**b**), the area is constantly isoechoic with respect to the surrounding liver parenchyma, as well as throughout the vascular study (arrow)

**Fig. 5:**
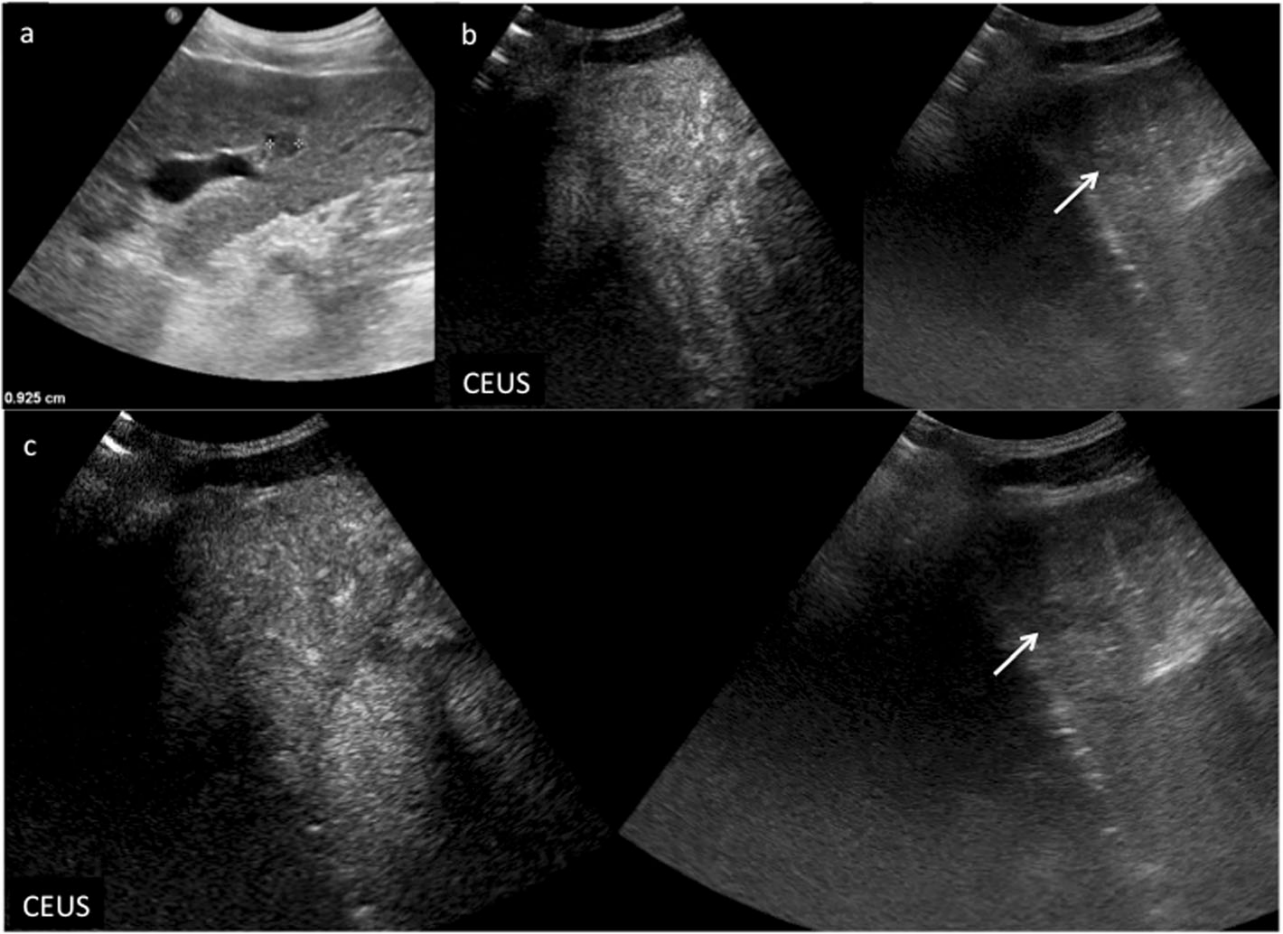
CEUS LI-RADS 2 (probably benign). Regenerative nodule in a 68-year-old woman with virus B-related cirrhosis. Baseline US image (**a**) shows a small hypoechoic lesion sized 0.9 cm in the segment III (calipers). At CEUS, the nodule is constantly isoechoic with respect to the surrounding liver parenchyma during the arterial (**a**) and extended portal venous phase (**b**, **c**)

**Fig. 6:**
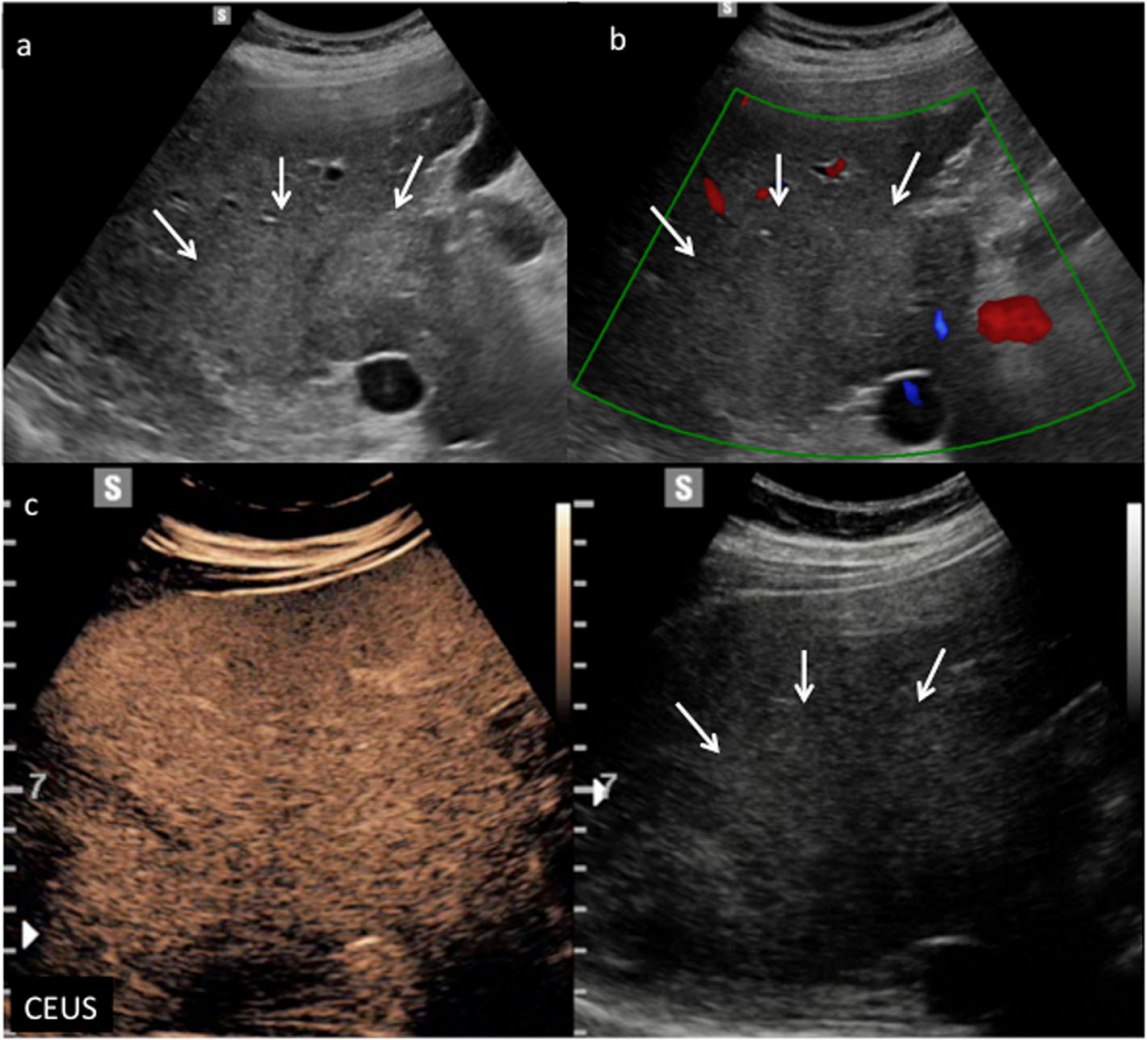
CEUS LI-RADS 2 (probably benign). Non mass-like area in a 57-year-old man with virus C-related cirrhosis. Baseline US image (**a**) shows a slightly hyperechoic area with indistinct margins sized 7 cm in the segment V–VI (arrows). The area does not show any vascular signal at color-Doppler (**b**) (arrows). At CEUS (**c**), it appears constantly isoechoic with respect to the surrounding liver parenchyma throughout the vascular study (arrows)

CEUS LR-1 and 2 categories include definitely and probably benign observations, respectively: in particular, LR-1 includes three main observation types: (1) cyst, (2) hemangioma, (3) fat deposition/sparing. A liver cyst is defined, as elsewhere in the body, as an anechoic lesion with increased posterior acoustic through transmission showing no contrast enhancement in any phase. Although simple cysts are easily detected and characterized even without the use of any contrast agent, CEUS may be of particular value in the characterization of more complex appearing lesions on B-mode US, showing their complete avascularity (Fig. 2) [8, 15]. Hemangioma is often recognized as a hyperechoic lesion, but it may present variable echogenicity on B-mode US [40]. CEUS easily depicts a typical peripheral globular enhancement in arterial phase followed by progressive centripetal fill-in and iso- or hyperenhancement in portal venous and late phase (Fig. 3) [41, 42]. The filling may be complete or partial depending on lesion size and/or the presence of mixoid or fibrotic degeneration [43]. Hepatic fat deposition/sparing is defined as nonmasslike, nonspherical, hyper/hypoechoic area of parenchyma in a characteristic location for fat deposition/sparing. Characteristic areas include liver parenchyma nearby the gallbladder and anterior to the right portal vein in segment 4. Hepatic fat deposition/sparing shows isoenhancement to the liver in all phases [44–46]. If the hyper/hypoechoic area is not in a characteristic location for fat deposition/sparing, categorize as CEUS LR-2 (see below) (Fig. 4). In case of detection of isoenhancing nodule at CEUS, observation should undergo CEUS LR-2 classification if solid nodule < 10 mm (Fig. 5), whereas if isoenhancing nodule is ≥ 10 mm, it should be categorized as CEUS LR-3 (see below). On the other hand, LR-3 nodules with interval size stability for more than 2 years can return to LR-2. These small nodules are probably typical regenerative or low-grade dysplastic nodules [47]. Any isoenhancing observation of any size, nonmasslike and without typical appearance of hepatic fat deposition or fat sparing, should be also categorized as LR-2 (Fig. 6).

Noteworthy, the CEUS enhancement features for HCC, hepatocellular adenoma (HCA,) and focal nodular hyperplasia (FNH) may overlap [47–54]. Hence, on a precautionary basis, in the clinical setting of patients at risk for HCC, nodules with CEUS feature of FNH and HCA should not be categorized as CEUS LI-RADS 1 or CEUS LI-RADS 2.

**Fig. 7:**
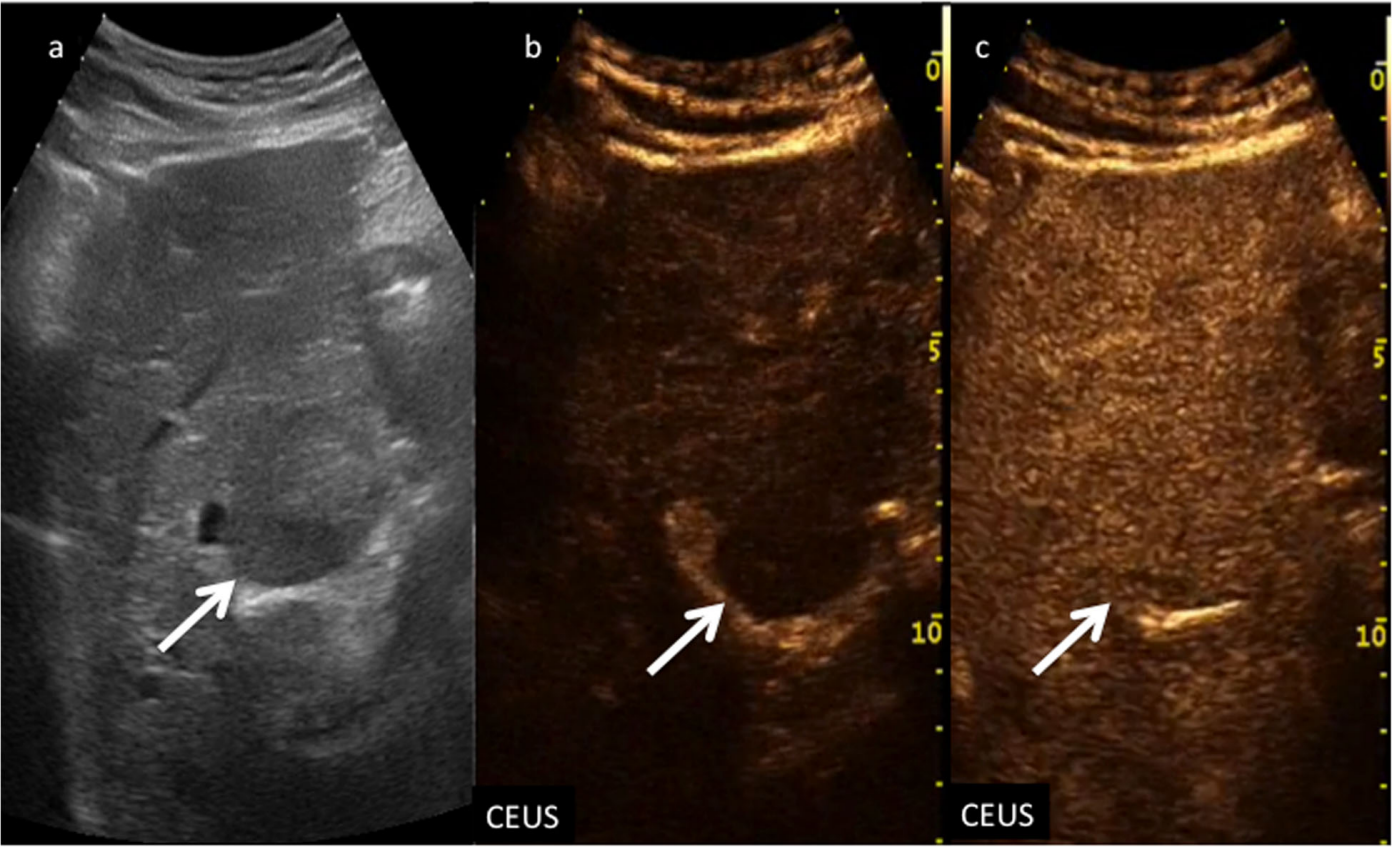
CEUS LI-RADS 3 (intermediate malignancy probability). Nodule in a 74-year-old man with virus B-related cirrhosis. Baseline US image (**a**) shows a slightly hypoechoic lesion sized 3 cm in the segment IV (arrow). At CEUS, the nodule appears moderately hypoechoic during the arterial phase (**b**) and isoechoic to the adjacent liver parenchyma in the extended portal venous (**c**) phase (arrows)

Any nodule not showing any APHE nor washout must be categorized as CEUS LR-3 regardless of size (Fig. 7). A nodule smaller than 2 cm, without any APHE but showing late and mild washout, should be also assigned to CEUS LR-3 category. On the other hand, any nodule larger than 2 cm, without any APHE but showing late and mild washout, must be assigned to CEUS LR-4 category.

**Fig. 8:**
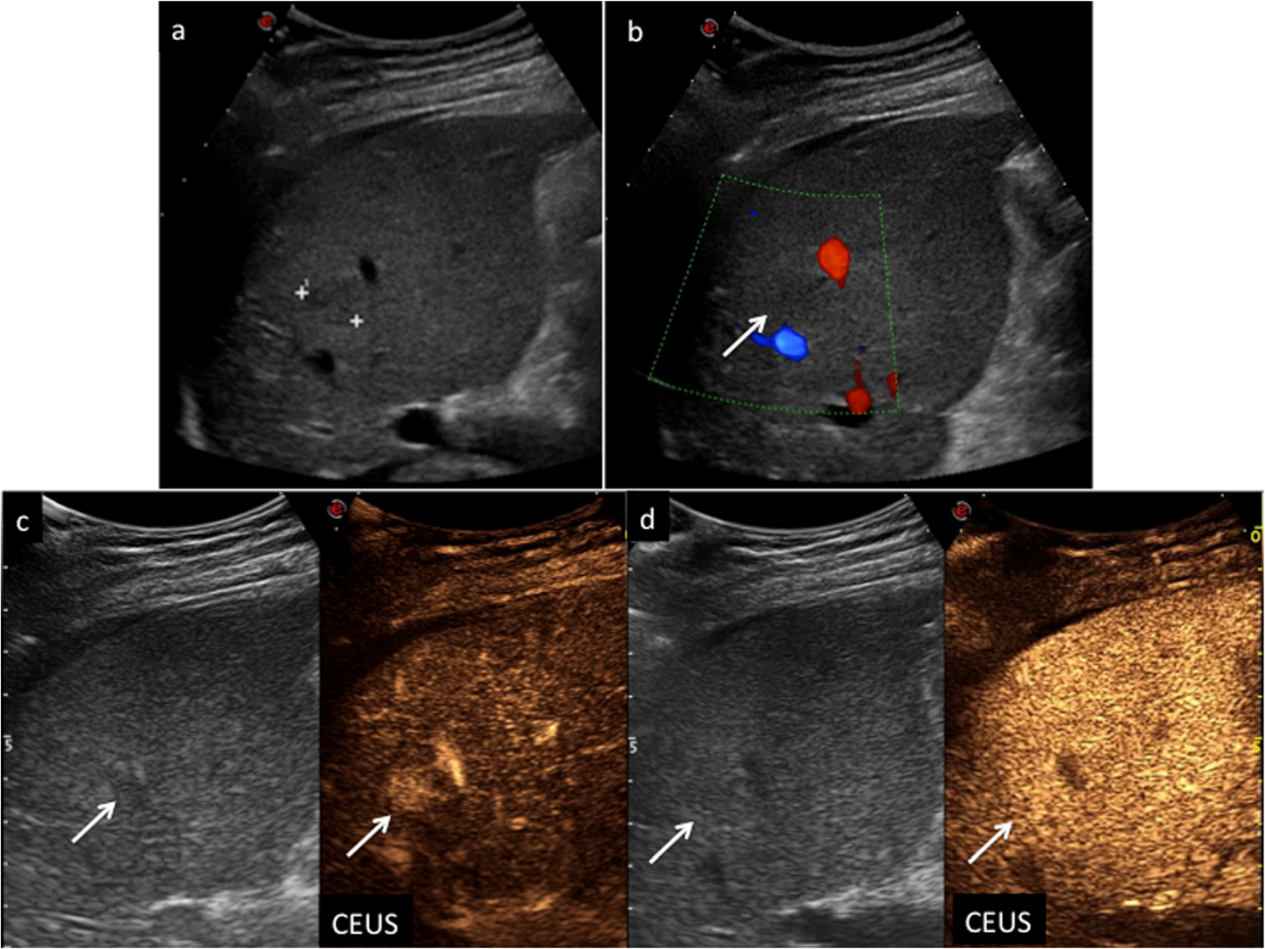
CEUS LI-RADS 4 (probably HCC). Nodule in a 52-year-old woman with virus C-related cirrhosis. Baseline US image (**a**) shows a slightly hypoechoic lesion sized 1.5 cm in the segment VIII (calipers). No vascular signal is detectable at color-Doppler (**b**) (arrow). At CEUS, the nodule is highly hypervascular during the arterial phase (**c**) (arrow) and isoechoic in the extended portal venous phase (**d**) (arrow)

**Fig. 9:**
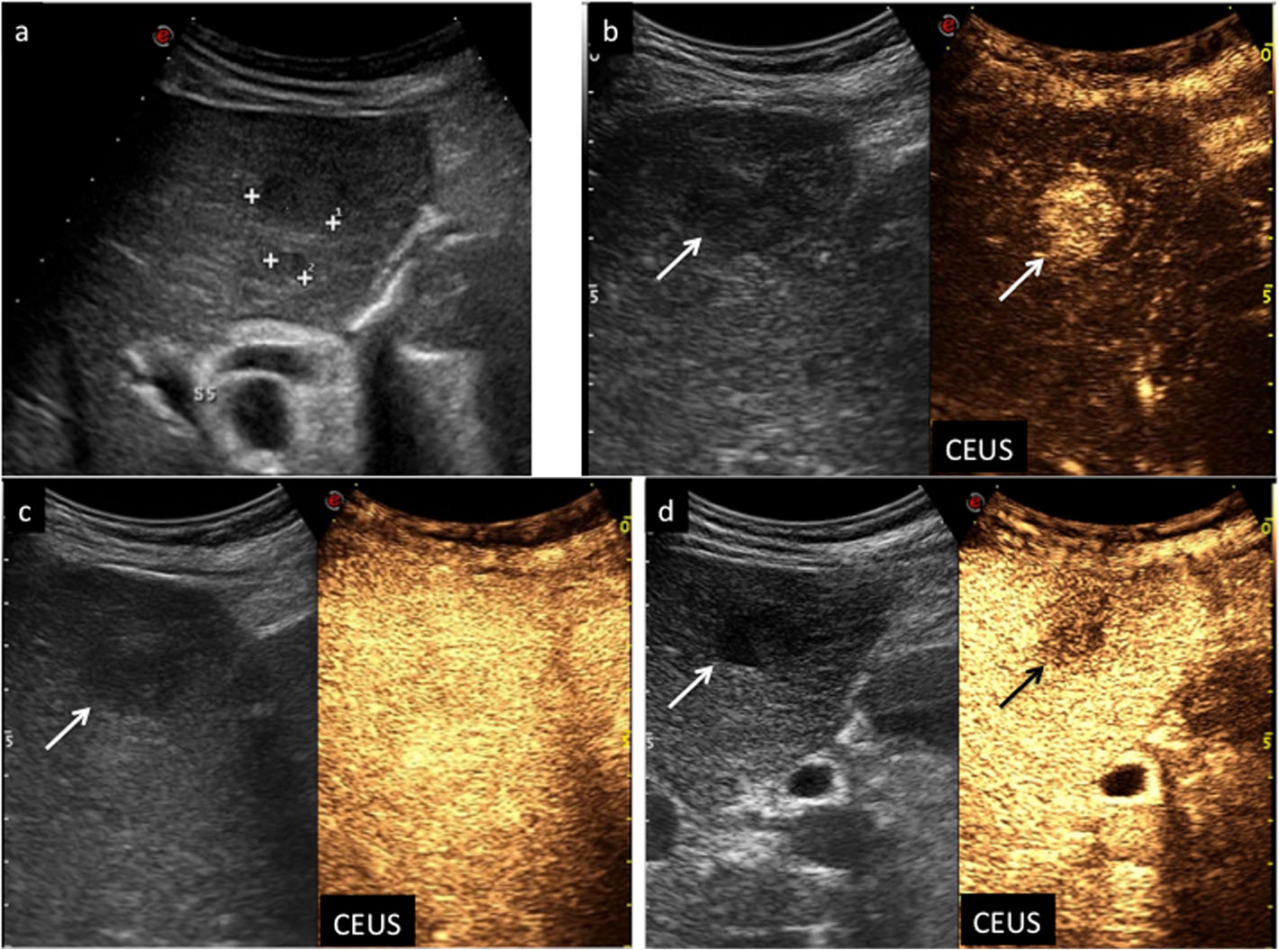
CEUS LI-RADS 5 (definitely HCC). Nodule in a 72-year-old man with virus B-related cirrhosis. Baseline US image (**a**) shows two moderately hypoechoic lesions sized, respectively, 2.2 cm and 0.7 cm in the segment V (calipers). At CEUS, the largest one is markedly hypervascular during the arterial phase (**b**) (arrows), becoming isoechoic 45 s after contrast medium injection (**c**) (arrow) and showing moderate wash-out 120 s after contrast medium injection (**d**) (arrows)

Nodules showing APHE (not rim or peripheral globular) without washout of any type should be categorized, depending on size, as CEUS LR-3 (when the nodule is smaller than 10 mm) or CEUS LR-4 (≥ 10 mm) respectively (Fig. 8). At the same time, nodules showing APHE (not rim or peripheral globular) but presenting with late and mild washout should be categorized, depending on size, as CEUS LR-4 (when the nodule is smaller than 10 mm) or CEUS LR-5 (≥ 10 mm) respectively (Fig. 9).

**Fig. 10:**
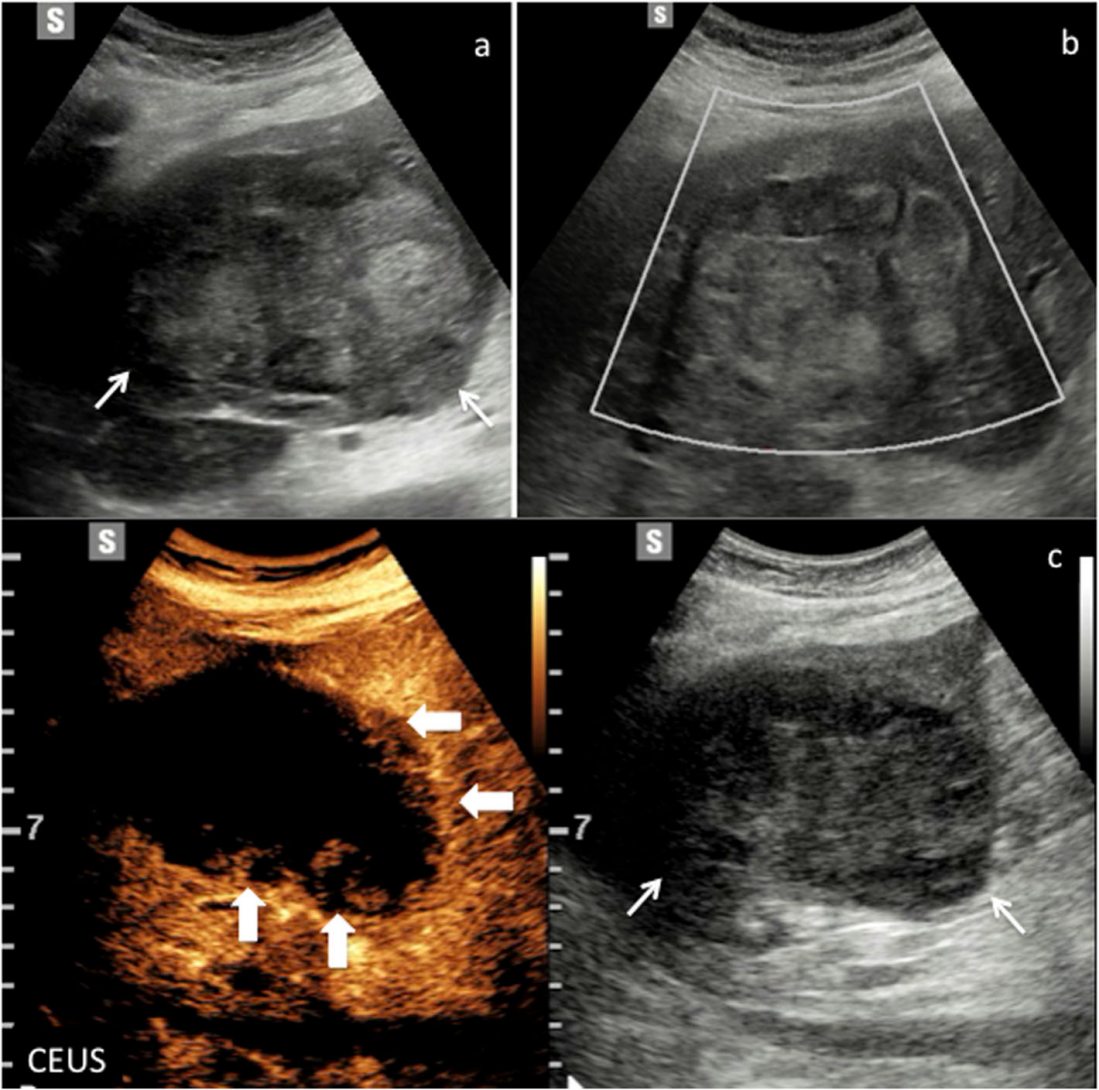
CEUS LI-RADS M (probably or definitely malignant but not HCC specific) nodule. Cholangiocarcinoma in a 87-year-old man with virus B-related cirrhosis. Baseline US image shows a markedly heterogeneous lesion sized 8.5 cm in the segment VI (**a**) (arrows). No vascular signal is detectable at color-Doppler (**b**). At CEUS (**c**), the nodule shows rim enhancement surrounding a constantly avascular area (arrows)

**Fig. 11:**
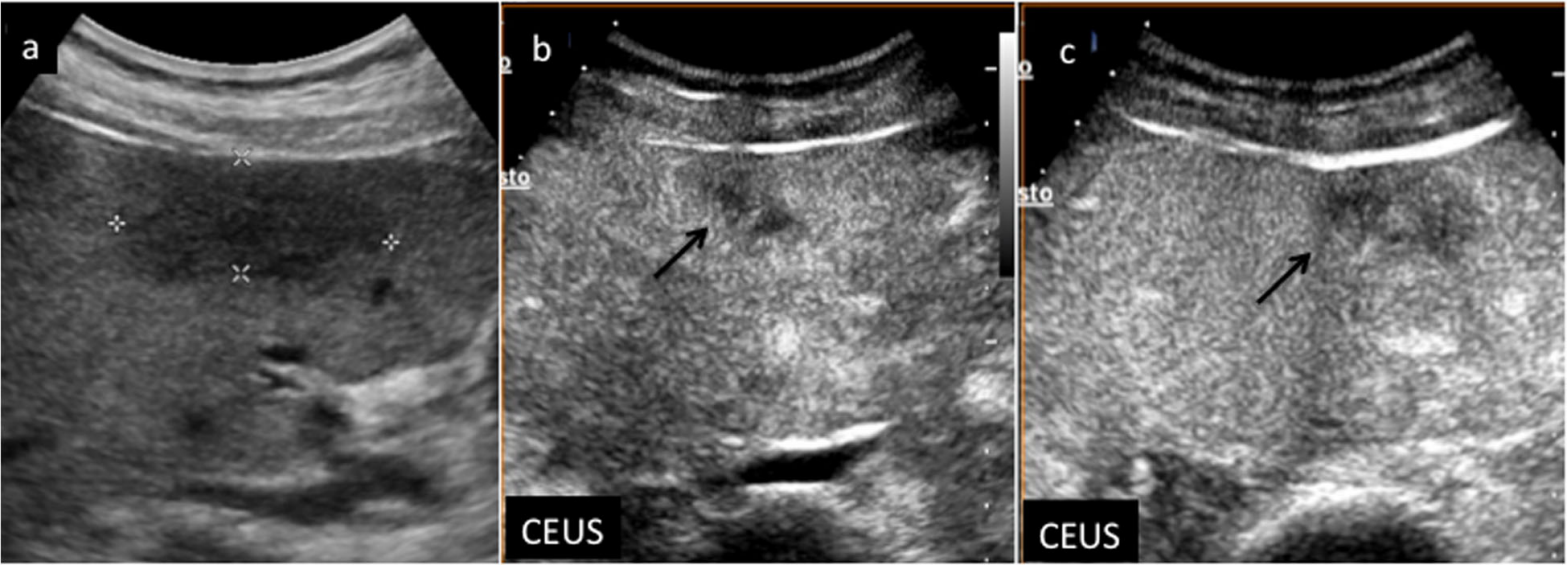
CEUS LI-RADS M (probably or definitely malignant but not HCC specific) nodule. Metastasis in a 32-year-old woman with virus B-related cirrhosis. Baseline US image (**a**) shows a hypoechoic lesion with not well-defined margins sized 5 cm in the segment IV (calipers). At CEUS, the lesion is heterogeneously vascularized during the arterial phase (**b**) (arrow) showing early wash-out: 34 s after contrast medium injection (**c**) (arrow)

In LR-M category, any nodule of any size should be included, which may show any of the following criteria [55–60]:

1. Rim APHE: “ring-like” APHE in which enhancement is most evident at the periphery of the nodule (Fig. 10);
2. Early (< 60 s) washout: temporally defined subtype of washout in which onset is within 60 s from contrast injection. Usually marked in degree (Fig. 11);
3. Marked washout.

At CEUS, the vast majority of malignant nodules typically show washout, including liver metastases, intrahepatic cholangiocarcinoma (ICC), and other tumors with fibrotic component which may show delayed central enhancement on CT or MRI [61–65]. Hence, to maintain specificity for HCC, CEUS characterization of washout requires assessment of its “onset” and “degree,” not just its presence. As consequence, observations with late and mild washout may be categorized as CEUS LR-3, LR-4, or LR-5. Nodules with early or marked washout should be categorized LR-M. ICC may be typically included in this category in a cirrhotic liver.

**Fig. 12:**
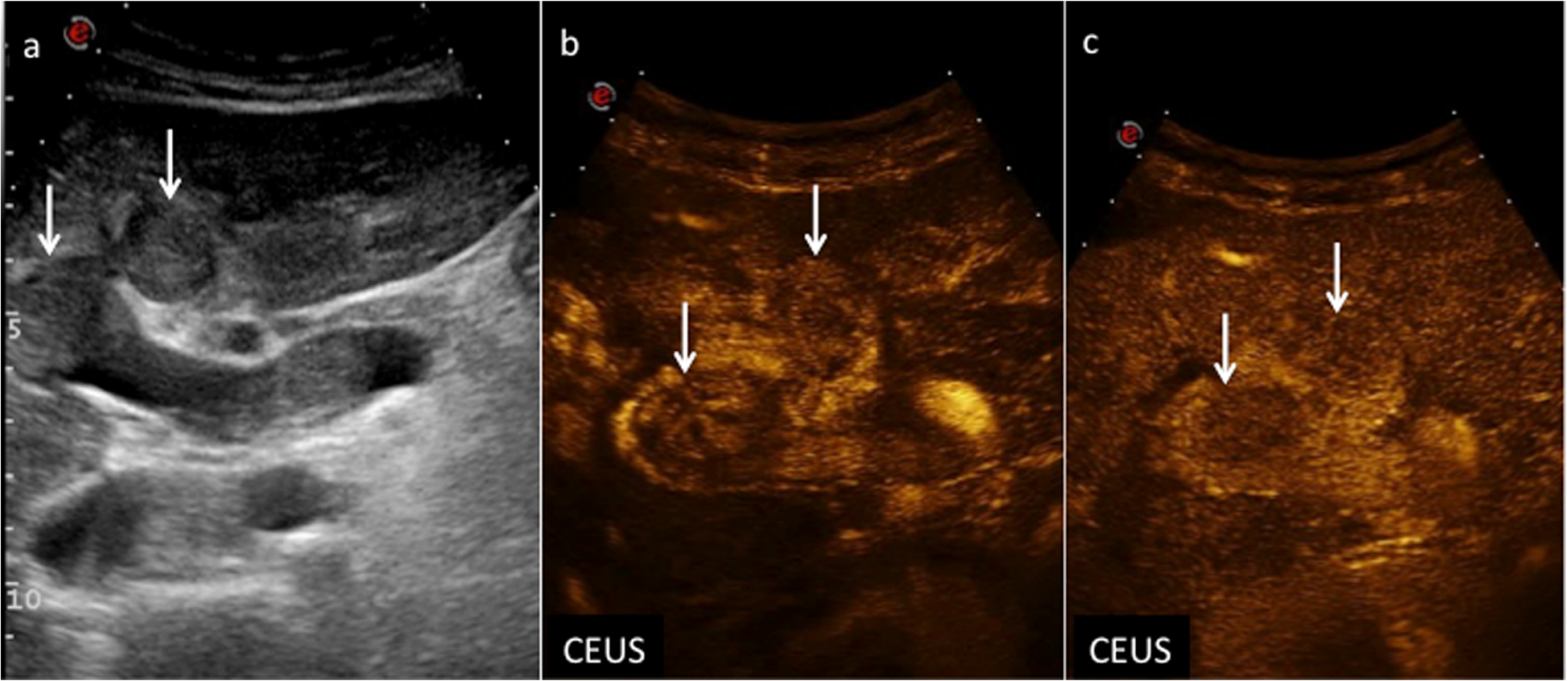
CEUS LI-RADS TIV (tumor in vein). Neoplastic thrombosis in a 84-year-old man with virus B-related cirrhosis. Baseline US image shows (**a**) multiple thrombi in the lumen of portal vein (arrows). At CEUS, marked enhancement is evident in their context during the early arterial phase (**b**) (arrows), followed by a clear-cut wash-out in the extended portal venous phase (**c**) (arrows)

Finally CEUS LR-TIV (definite tumor in vein) includes observation of enhancing tissue within a vein, independently from the detection of a coexisting liver tumor. Tumoral invasion of veins must be differentiated from bland thrombus [66]. To this purpose, the arrival time of microbubble contrast agent to the vein helps to distinguish tumor in vein from bland thrombus [67]:

- Early arrival time (~ same time as contrast enhancement of hepatic artery): favors tumor in vein (Fig. 12).
- Arrival time of several seconds (~ 10) after contrast enhancement of hepatic artery: favors portal flow in patent portion of non-occlusive/recanalized bland thrombus.

**Fig. 13:**
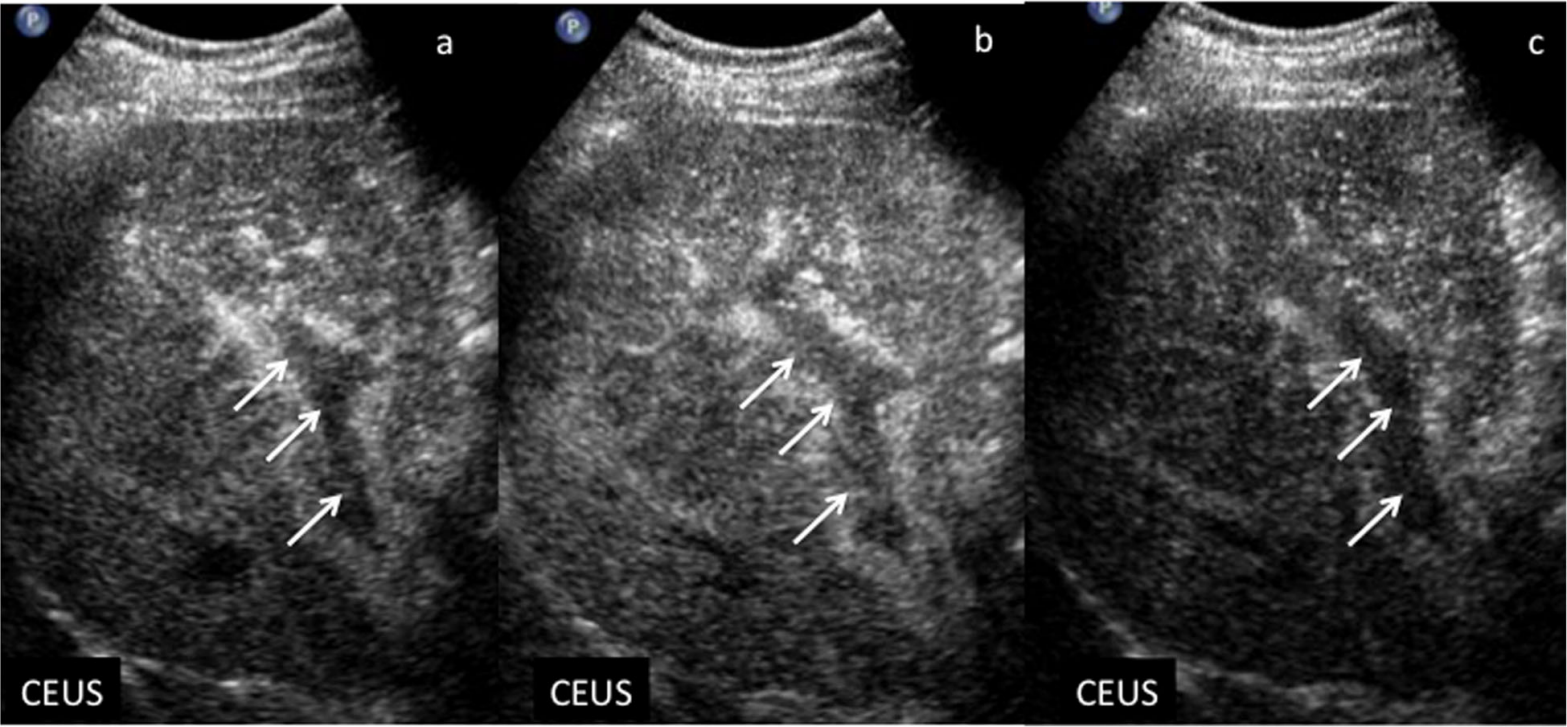
Non-neoplastic thrombosis in a 62-year-old man with virus B-related cirrhosis. At CEUS, no enhancement is appreciable in the context of the right branch of portal vein during the arterial (**a**), portal venous (**b**), and late (**c**) phases, respectively (arrows)

Bland thrombus shows lack of vascularization (Fig. 13). The proximity with any liver mass may help in etiology definition. In particular, if TIV is contiguous or associated with any LI-RADS 4 or 5 lesions, tumor in vein is probably or definitely attributable to HCC, whereas if TIV is near LR-M, it is probably due to non-HCC malignancy. If no masses are detected, etiology is undetermined.

### HCC and CEUS LI-RADS: final considerations

CEUS is currently recommended as an adjunct tool in the imaging work-up of HCC, either in the LI-RADS lexicon or in other international guidelines, with encouraging results also in terms of cost-benefit analysis [24, 68, 69]. Of note, there is still lack of consensus among different Scientific Societies regarding the precise role of CEUS in the diagnostic algorithm for the characterization of HCC. On one hand, various scientific societies, including ACR and Japanese, Italian, German, and British, suggest the use of CEUS in the diagnostic algorithm of HCC in their guidelines (www.webaisf.org, www.drg.de, and www.nice.org.uk, respectively). In the latest version of European Association for Study of Liver (EASL) guidelines on the management of HCC, CEUS is also considered a diagnostic tool for HCC as well as CT and MRI [68]. On the other hand, other Korean and American Societies, such as the Korean Liver Cancer Study Group, the American Association for the Study of Liver Diseases, and the Organ Procurement and Transplantation Network, suggest the use of CT and MR only [68, 69]. Further refinement may allow a desirable and better uniformity in international guidelines.

## Data Availability

Not applicable.

## Abbreviations

ACR: American College of Radiology
AP: Arterial phase
APHE: Arterial phase hyperenhancement
CEUS: Contrast-enhanced ultrasound
CT: Computed tomography
FFLs: Focal liver lesions
FNH: Focal nodular hyperplasia
HA: Hepatocellular adenoma
HBV: Hepatitis B virus
HCC: Hepatocellular carcinoma
HCV: Hepatitis C virus
ICC: Intrahepatic cholangiocarcinoma
LI-RADS: Liver Imaging Reporting and Data System
LP: Late phase
MDD: Multidisciplinary discussion
MRI: Magnetic resonance imaging
NAFLD: Nonalcoholic fatty liver disease
PVP: Portal venous phase
US: Ultrasound
USCAs: US contrast agents
